# Transmissive-detected laser speckle contrast imaging for blood flow monitoring in thick tissue: from Monte Carlo simulation to experimental demonstration

**DOI:** 10.1038/s41377-021-00682-8

**Published:** 2021-12-03

**Authors:** Dong-Yu Li, Qing Xia, Ting-Ting Yu, Jing-Tan Zhu, Dan Zhu

**Affiliations:** 1 Britton Chance Center for Biomedical Photonics, Wuhan National Laboratory for Optoelectronics, Huazhong University of Science and Technology, 430074 Wuhan, Hubei China; 2 MoE Key Laboratory for Biomedical Photonics, Huazhong University of Science and Technology, 430074 Wuhan, Hubei China

**Keywords:** Imaging and sensing, Biophotonics

## Abstract

Laser speckle contrast imaging (LSCI) is a powerful tool to monitor blood flow distribution and has been widely used in studies of microcirculation, both for animal and clinical applications. Conventionally, LSCI usually works on reflective-detected mode. However, it could provide promising temporal and spatial resolution for in vivo applications only with the assistance of various tissue windows, otherwise, the overlarge superficial static speckle would extremely limit its contrast and resolution. Here, we systematically investigated the capability of transmissive-detected LSCI (TR-LSCI) for blood flow monitoring in thick tissue. Using Monte Carlo simulation, we theoretically compared the performance of transmissive and reflective detection. It was found that the reflective-detected mode was better when the target layer was at the very surface, but the imaging quality would rapidly decrease with imaging depth, while the transmissive-detected mode could obtain a much stronger signal-to-background ratio (SBR) for thick tissue. We further proved by tissue phantom, animal, and human experiments that in a certain thickness of tissue, TR-LSCI showed remarkably better performance for thick-tissue imaging, and the imaging quality would be further improved if the use of longer wavelengths of near-infrared light. Therefore, both theoretical and experimental results demonstrate that TR-LSCI is capable of obtaining thick-tissue blood flow information and holds great potential in the field of microcirculation research.

## Abstract (web summary)

The performance of novel transmissive-detected LSCI was systematically demonstrated through simulation and experiments. With such a simple system, individual vessel-resolution blood flow mapping and monitoring were realized on human hand.

## Graphical abstract

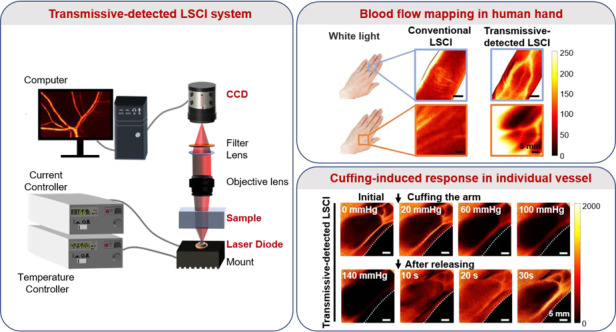

## Introduction

Laser speckle contrast imaging (LSCI) is a wide-field, noninvasive imaging technique with high temporal and spatial resolution, which is based on the analysis of light signals after scattering and random interference, and therefore obtains the velocity information of scattering particles in biological tissues (e.g., red blood cells)^1^. Conventionally, it works on the reflective-detected mode, and has been widely used in the fundamental research of microcirculation^2–7^, whose dysfunction is highly relevant to a series of clinical symptoms, such as diabetes^8^, ischemic stroke^9,10^, coronary heart disease^11^ and peripheral artery disease^12^. With surgery-based open-skull windows, thinned-skull windows, and surgery-free skull optical clearing windows, cortical blood flow distribution could be clearly observed using conventional reflective-detected LSCI technique^13^. With skinfold chamber windows and skin optical clearing windows, conventional LSCI could also provide cutaneous blood flow mapping with individual-blood-vessel resolution^14^. However, without such “windows”, the light should penetrate the upper tissue layer above the deep blood vessel layer, during which path it constantly decays, making the strength of static speckle in the upper layer much greater than that of dynamic speckle signal in the deep targeted layer, leading to the extremely decreased contrast and resolution of conventional LSCI, or even making the blood flow undetectable^15^. Moreover, even with the assistance of skull and skin windows, conventional LSCI is still only able to provide acceptable resolution in the superficial layers, while even the body parts of mice are often hundreds of microns or even millimeters thick, making it barely possible to obtain comprehensive information using such a technique^16–18^.

In order to overcome such a problem, researchers further looked to algorithms for solutions, such as calculating temporal contrast instead of spatial contrast analysis to reduce the effect of static speckle^19^, while the imaging quality improvement was still limited. Later, there were preliminary results which indicated that changing the direction of incident light might contribute to the extraction of thick-tissue information^20,21^. It was observed that transmissive-detected laser speckle contrast imaging (TR-LSCI) showed better imaging contrast and resolution than conventional LSCI on blood flow mapping in the hindlimb of mice and could even provide microvascular functional imaging in human finger joints without other supplementary means. However, such discoveries have not got many attentions, and the TR-LSCI technique has not been thoroughly studied, therefore the mechanisms why TR-LSCI might be promising for blood flow monitoring in thick tissue remain unclear. What is the effect of the depth and thickness of the target tissue on these two imaging modes? What are the factors those could influence the performance of TR-LSCI? Is there a situation where the imaging quality of conventional LSCI is better than that of TR-LSCI? The answers to these questions will enrich the theory of laser speckle imaging for biological tissues.

This work aims to systematically evaluate the capability of TR-LSCI for thick-tissue observation from theory to practice. Here, Monte Carlo technique^22^, a powerful tool to simulate the optical propagation and distribution in the tissue, was used to reveal the law of the signal-to-background-ratio (SBR) of reflective- and transmissive-detected imaging modes with varying signal depth and tissue thickness, mechanically illustrating why TR-LSCI is more suitable for thick-tissue imaging. Next, a series of experiments on 3 levels, including tissue phantom level, animal level, and human level, were designed to evaluate the validity of the Monte Carlo simulation results. The two imaging modes were firstly compared in the intralipid with the change of thickness and signal depth. Secondly, cutaneous/subcutaneous blood flow mapping in different mice body parts (ear, hindlimb, back and paw) was performed. Finally, conventional LSCI and TR-LSCI were applied in human hand imaging (finger and opisthenar). All the experimental results well fit the conclusion of the Monte Carlo simulation. Therefore, this work compared the performance of conventional LSCI and TR-LSCI when used in bioimaging and provides a guidance for researchers to choose a proper imaging technique to achieve better imaging quality in thick tissue, which is significant in in vivo microcirculation studies.

## Results

### Monte Carlo simulation for theoretical analysis

**Fig. 1:**
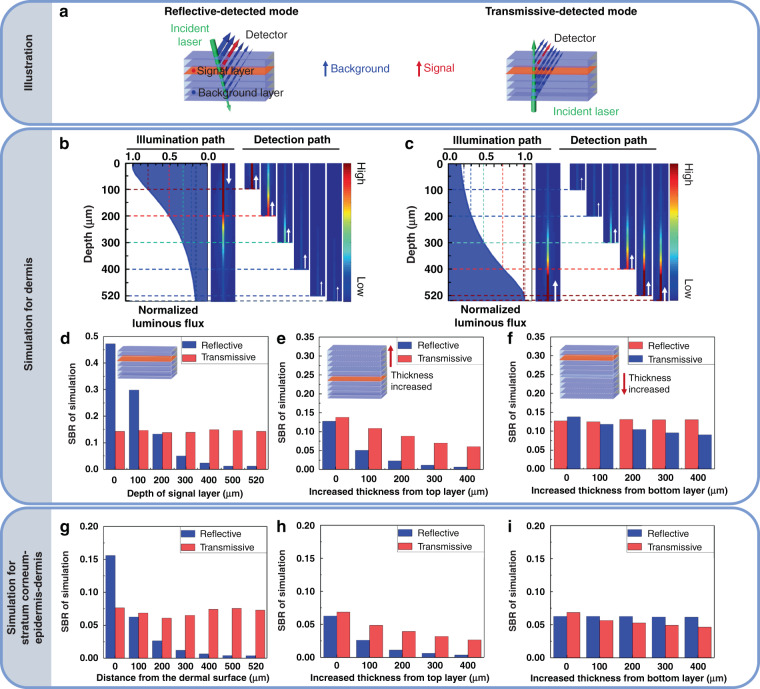
Monte Carlo simulation. **a** Schematic diagram of the distribution of the signals in reflective and transmissive modes, respectively. The green arrows represent the attenuation of the light source when penetrating the tissue (set as 520 μm, divided into 7 layers). The red layers and arrows represent the signals from the interested layers. The blue layers and arrows represent the signals from other layers. The thickness of the arrow represents the intensity of signal. **b**, **c** The normalized luminous flux over depth for quantitative analysis of attenuation of light in the simulated tissue for reflected-detected imaging (**b**) and transmissive-detected imaging (**c**). The white arrows indicate the direction of light propagation. The zoom-in regions of (**b**) and (**c**) represent the light path from the certain depth to the surface of the tissue, where the color represents the light flux intensity distribution in the light path, and the data were normalized to the light flux when the illumination light first accessed the tissue, where the light first accessed to the surface for reflective-detected mode and first accessed to the bottom of the tissue for transmissive-detected mode. **d** SBR of two modes when the depth of signal layer was set as 0, 100, 200, 300, 400, 500, 520 μm, respectively. **e**, **f** SBR with the increased thickness from top layer (**e**) and bottom layer (**f**) while the depth of signal layer was fixed as shown in (**a**). **g** SBR of two modes when the depth of signal layer was set as 0, 100, 200, 300, 400, 500, and 520 μm of the simulated dermis, respectively, while 80 μm of epidermis and 20 μm of stratum corneum were successively above the dermis. **h**, **i** SBR with the increased thickness from top layer (**e**) and bottom layer (**f**) of dermis while the depth of signal layer is fixed as 100 μm to the surface of dermis in (**g**). Here, SBR was defined as the proportion of light emanating from the target layer to the total light emanating from each layer

Figure 1 shows the results of the Monte Carlo simulation. Firstly, a single-component tissue with a thickness of 520 μm was set up to simulate the dermis. As shown in Fig. 1a–c, the incident light continually decayed as it penetrated the tissue, therefore the closer layer to the incident point would get larger luminous flux (the surface was twice as strong as it was at 200 μm). As shown in Fig. 1b, for reflective mode, the illumination light accessed the tissue at the surface, and the fluxes at different layers decreased from top to bottom of the tissue. Consequently, the superficial layer could generate more signal, Next, the signals at each depth would get back to the surface so they could be captured by the detector. In this process, the signals from the superficial layer suffered less attenuation to penetrate the tissue to access the detector than that of the deep layer (the zoom-in region of Fig. 1b). Therefore, the SBR at the superficial layer was promising but would decay rapidly as the signal depth increased (Fig. 1d). On the contrary, for transmissive mode, the illumination light accessed the tissue at the bottom. Therefore, although the superficial layer suffered less attenuation to access the detector, the initial signal intensity was much less than that of the deep layer, leading to relatively stable SBR at various depths (Figs. 1c and 1d). The results indicated that reflective mode was better for superficial-tissue imaging, while transmissive mode was preferential for deep-tissue imaging at a certain thickness. In our simulation with certain settings (520-μm thickness), the SBR of two imaging modes were comparable at 200 μm. On this basis, we simulated the change of SBR when the upper or lower layer of the tissue was thickened. As shown in Fig. 1e, the thickening of the upper layer had a greater effect on the SBR in reflective mode. When the upper layer was increased by 200 μm, the SBR of reflective mode decreased by 82% while the SBR of transmissive mode only decreased by 36%. When the upper layer was increased by 400 μm, the SBR of the transmissive mode decreased to 44% of the initial value while the SBR of the reflective mode only remained 4%. Figure 1f suggested that the thickening of the lower layer had a greater effect on the SBR in transmissive mode while showed no remarkable influence on the SBR of reflective mode. Comparison of Fig. 1e and Fig. 1f showed that upper layer thickening had a greater effect on both imaging modes. Furthermore, we simulated a tissue with stratum corneum (20 μm) and epidermis (80 μm) above the dermis (520 μm) because it was closer to the actual biological case. We analyzed the SBR when the detected signal was at different depths of dermis. Similar to the simulation results of the single-component tissue, the SBR of transmissive mode was much more stable than that of reflective mode (Fig. 1g), and the influence of the thickening of the upper or lower layer on the SBR of reflective and transmissive mode respectively was also consistent with the single-component tissue (Figs. 1h, i).

### Blood flow mapping of tissue phantom with conventional LSCI and TR-LSCI

**Fig. 2:**
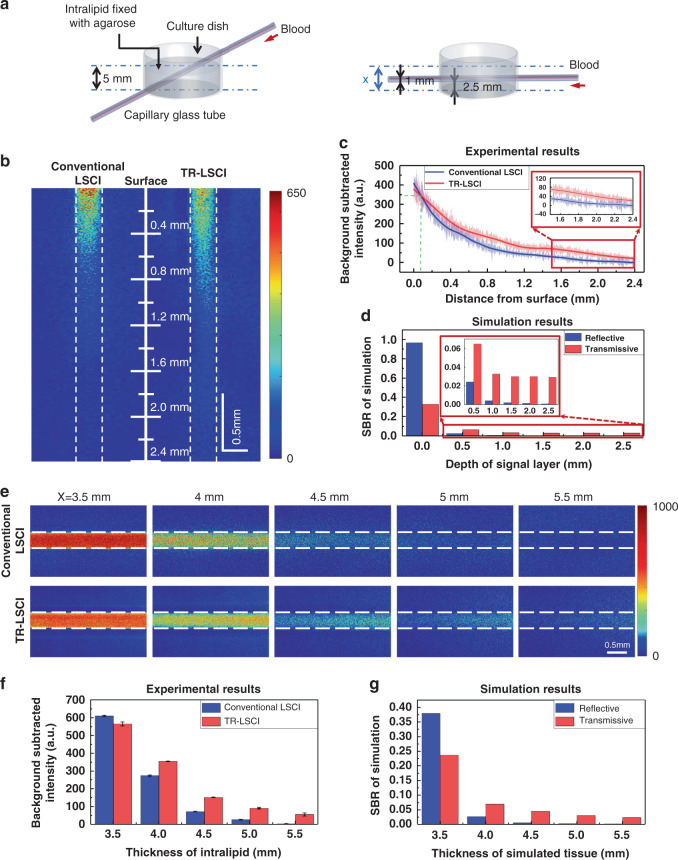
Imaging for tissue phantom. **a** Schematic diagram of tissue phantom experimental devices. The experiments consisted of two parts: 1. The total thickness was constant, and the blood flow signals at /from different depths were measured (left); 2. The position of the blood flow signal was constant, and the thickness of the layer above the signal was changed (right). **b** Blood flow maps of part 1 captured with conventional LSCI and TR-LSCI (The dashed lines represent the inner wall of the capillary glass tube). The scale line represents the distance to the surface of the intralipid. The blood was pumped to flow in the capillary glass tube at a constant speed of 4 mm s^−1^. **c** Quantitative analysis of effect of the depth of the capillary on background subtracted signal intensity. Data are expressed in (mean ± standard error), *n* = 3. **d** SBR of two imaging modes in the Monte Carlo simulation according to the corresponding settings in (**c**). Here, SBR was defined as the proportion of light emanating from the signal layer to the total light emanating from each layer. **e** Blood flow maps of part 2 captured with conventional LSCI and TR-LSCI (The dashed lines represent the inner wall of the capillary glass tube). The pump pushes the blood to flow in the capillary glass tube at a constant speed of 4 mm s^−1^. **f** Quantitative analysis of effect of the thickness of the intralipid on background subtracted signal intensity. Data are expressed in (mean ± standard error), *n* = 3. **g** SBR of two imaging modes in the Monte Carlo simulation according to the corresponding settings in (**e**). Here, the SBR was defined as the proportion of light emanating from the signal layer to the total light emanating from each layer

The construction of tissue phantom where capillary glass tube was in the intralipid could be used to simulate blood vessel at different depths in biological tissues or blood vessel in tissue of different thicknesses (Fig. 2a). As shown in Fig. 2b, when the thickness of the intralipid was set as 5 mm and stayed constant, the performance of conventional LSCI at the surface was better than that of TR-LSCI, but was worse at large depth, which is consistent with the conclusion in Fig. 1d. Further quantitative analysis showed that TR-LSCI surpasses conventional LSCI with a depth of 80 μm. Figure 1d shows that the imaging performance of TR-LSCI would not obviously decrease with the signal went deeper, which seemed not to fit the experimental results here. Since the simulated tissue thickness was only set as 520 μm in Fig. 1, we performed Monte Carlo simulation again, with the simulated tissue of 5 mm. This time, it was found that the performance of TR-LSCI would also reduce with the signal depth, but always better than that of conventional LSCI for deep-tissue imaging (Fig. 2d), which fit the experimental result well. In addition, under the experimental conditions of constant signal position and increasing the upper tissue phantom, it was also found that compared to conventional LSCI, TR-LSCI was less sensitive to the increase in thickness of the upper layer (Figs. 2e, f), which also verified the feasibility of Monte Carlo simulation (Fig. 2g). Furthermore, if the intralipid with a capillary at the superficial layer rotated 180°, the SBR of TR-LSCI would not degrade as much as conventional LSCI (Fig. S1). Moreover, since a polarizing filter is a critical component of a LSCI system, which could contribute to the imaging,^23^ we also performed comparison between conventional LSCI and TR-LSCI with a polarizing filter matching the light source in front of the detector. The results indicated that, the polarizing filter could indeed improve the performance of both two modes. In addition, the comparison between two imaging modes was similar to that without the polarizing, where TR-LSCI was comparable at surface, and performed much better at large depth. (Fig. S2).

**Fig. 3:**
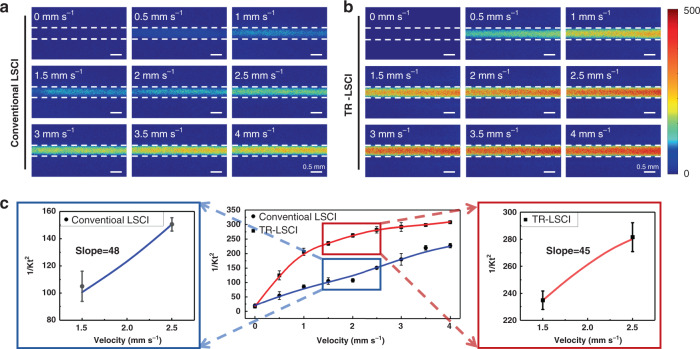
Comparison of blood flow sensitivity between conventional LSCI and TR-LSCI. **a** Representative conventional LSCI images with various blood flow velocity. **b** Representative TR-LSCI images with various blood flow velocity. **c** Plots of 1/K_t_^2^ to velocities for conventional LSCI and TR-LSCI. *K*_t_ was the temporal contrast. Data are expressed in (mean ± standard error)

The sensitivity of conventional LSCI and TR-LSCI to flow changes was further compared. A capillary was put inside a 4.5-mm-thick intralipid (1 mm from the surface, 2.5 mm from bottom), and the blood flow velocity was set from 0 to 4 mm s^−1^. As shown in Figs. 3a, b, the signal intensity of TR-LSCI for the blood flow in the capillary was higher than that of conventional LSCI at various velocity. The quantitative analysis shown in Fig. 3c indicated that the sensitivity to flow change of TR-LSCI decreased as the velocity increased. However, at lower velocity (<2 mm), the sensitivity of TR-LSCI was obviously higher than that of conventional LSCI. Such results demonstrated TR-LSCI held advantages not only in deep-tissue blood flow mapping but also in the sensitivity to blood flow change at a small velocity.

Here, the speckle sizes of conventional LSCI and TR-LSCI were calculated to be similar in our experimental condition (2.50 pixels vs. 2.45 pixels with ×2 magnification). Since it has been demonstrated that the speckle size would influence the sensitivity of conventional LSCI to the change of velocity, it is valuable to explore the influence of speckle size on TR-LSCI. As shown in Fig. S3, the temporal contrast increased as the speckle size increased at the same flow velocity, and higher speckle size would enhance the flow change sensitivity of TR-LSCI, which was consistent with the results of conventional LSCI in the previous studies^24^.

### Blood flow mapping of body parts in mice with conventional LSCI and TR-LSCI

**Fig. 4:**
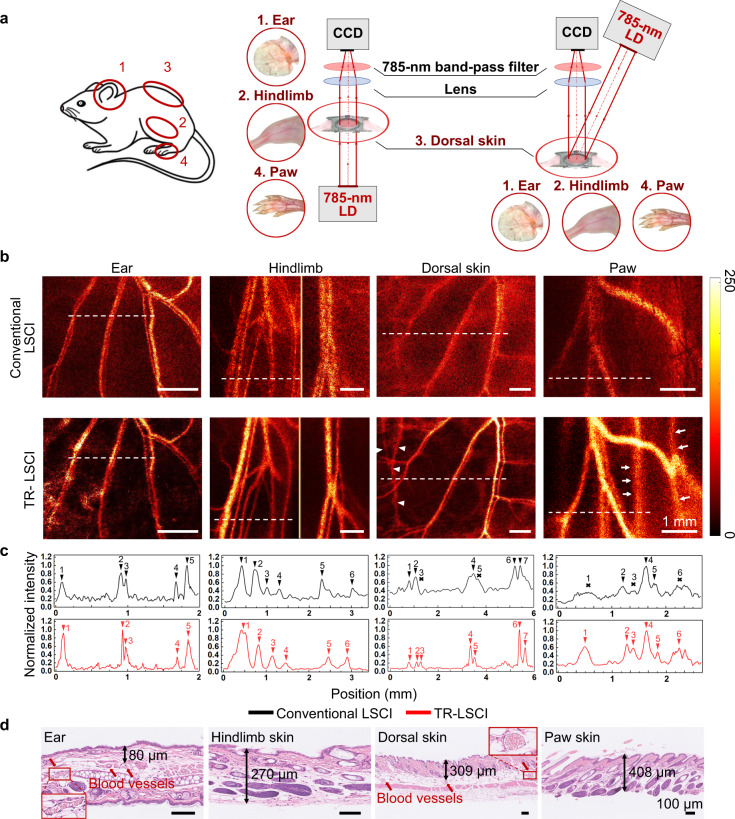
Comparison of conventional LSCI and TR-LSCI for blood flow mapping in ear, hindlimb, back and paw of mice. **a** Illustration of the system used for LSCI for ear, hindlimb, back and paw of mice. **b** Typical blood flow maps of cutaneous/subcutaneous vessels imaged by conventional LSCI and TR-LSCI. The white arrow heads indicate opposite dorsal subcutaneous blood vessels, and the white arrows indicate the blood vessels between tendons and metatarsals in the paw. **c** Line plots of dashed lines in (**b**). Black arrows indicate the position of vessels, and the crosses indicate the unidentifiable blood flow. **d** H&E staining of ear, skin on hindlimb, back and paw for measuring tissue thickness above the blood vessels. Red arrows indicate blood vessels containing red blood cells in ear and dorsal skin. The imaged blood vessels in mice hindlimb and paw were under the skin. The marked length is the thickness of skin tissue after H&E staining, which does not represent the real situation in vivo, and is only used to reflect the relative thickness of skin tissue in different body parts

For in vivo comparing conventional LSCI and TR-LSCI, cutaneous/subcutaneous blood flow mapping in the ear^25^, hindlimb^26^, dorsal skin^27^, and paw^7^ of mice (Fig. 4a) was performed, where the microcirculation was always studied. For TR-LSCI, the light source was put under the mice body parts, respectively, and the detector was put above the mice. For conventional LSCI, the detector was at the same position as TR-LSCI, while the illumination light diagonally irradiated on the surface of the mice body parts. Therefore, in this case, the light detected path for two imaging modes were the same, and the “superficial layer” was always defined as the layer close to the detector. As shown in Fig. 4b, c, the imaging quality of conventional LSCI was poor except for the ear, which was just acceptable. In contrast, the SBR of TR-LSCI was higher than that of conventional LSCI in every ROI (also see Table 1). In addition, it was found that the imaging quality of TR-LSCI for ear was the best, while it was the worst for the paw. Figure 3d shows that the depth of blood vessels in mice ear was the smallest, while the depth of the blood vessels in mice paw was the largest. Such experimental results well matched the Monte Carlo simulation results that the influence of upper thickness of target signal on transmissive-detected imaging mode was greater than that of lower thickness.

**Table 1:** SBR of blood vessels in mice ear, hindlimb, back, and paw

Part	Mode	1 #	2 #	3 #	4 #	5 #	6 #	7#
Ear	Conventional LSCI	2.72	3.74	3.31	2.99	3.70		
	TR-LSCI	10.45	11.40	7.70	4.66	7.53		
Hindlimb	Conventional LSCI	3.73	2.80	1.51	1.91	2.47	1.58	
	TR-LSCI	10.18	7.65	4.73	3.11	4.40	5.14	
Back	Conventional LSCI	1.66	1.79	–	2.00	–	2.26	2.20
	TR-LSCI	3.20	2.79	3.15	8.24	4.28	12.87	7.36
Paw	Conventional LSCI	–	1.89	–	2.97	1.74	–	
	TR-LSCI	2.82	3.11	1.86	3.18	2.06	2.43	

“–” indicates the blood flow signal could not be distinguished from the background.

Moreover, we found that using TR-LSCI, it was able to observe information of blood vessels at different depths in the paw, including subcutaneous blood vessels and deeper blood vessels between tendons and metatarsals (white arrows in Fig. 4b). Similarly, when the subcutaneous blood vessels in one side of the skinfold chamber were imaged with TR-LSCI, the subcutaneous blood flow from the other side was acquired at the same time (white arrow heads in Fig. 4b). Thus, we could obtain more comprehensive blood flow mapping relying on TR-LSCI.

### Ach-induced vascular functional response in mice hindlimb monitored with TR-LSCI

**Fig. 5:**
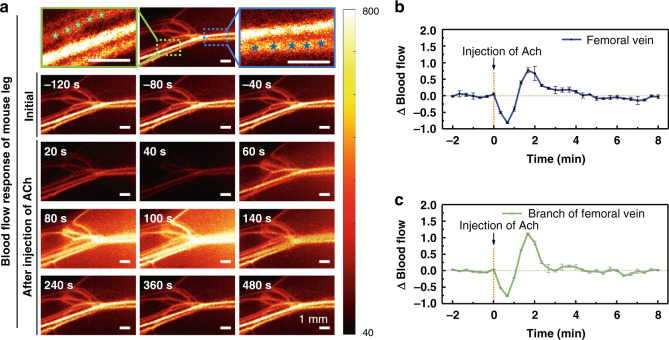
Maps of dynamic response of blood flow in the hindlimb of mice. **a** Typical blood flow maps of subcutaneous blood vessels before and after the injection of Ach. The blue and green asterisks state the femoral vein and its branch. **b**, **c** Statistical analysis based on blood vessel positions indicated by asterisks in the blue and green solid line rectangular boxes was performed to show the relative changes in blood flow of femoral vein (**b**) and its branch (**c**) over time. Data are expressed in (mean ± standard error)

The capability of TR-LSCI on noninvasively tracking subcutaneous vascular functional response was evaluated in mice. The reaction of mice hindlimb vascular blood flow to acetylcholine (Ach) was presented in Fig. 5a. The femoral artery, femoral vein, and their branches could be clearly identified. As shown in Fig. 5b, c, the relative changes in blood flow of the femoral vein and its branch were analyzed quantitatively. Before injection, the blood flow kept stable. After Ach was injected, the blood flow in both femoral vein and its branch decreased rapidly and reached the bottom (10% of that baseline value) at 40 s, after which blood flow continued to rise to a peak. Blood flow of femoral vein rise to 180% of baseline value and recovered to initial level after 160 s. However, blood flow of branch increased to 220% and it took 80 s to recover. The results indicated TR-LSCI provided both individual vascular structural and functional information.

### Blood flow mapping of human hand with conventional LSCI and TR-LSCI

**Fig. 6:**
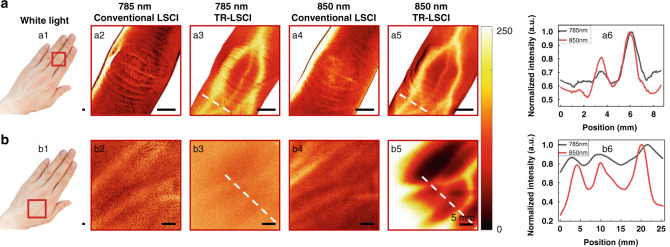
Comparison of conventional LSCI and TR-LSCI for blood flow mapping in human hand. **a** Images of subcutaneous blood vessels in the middle finger. **a1** photograph of human hand. Red rectangular box indicates the ROI. **a2**–**a3** Conventional LSCI (**a2**) and TR-LSCI (**a3**) for blood flow mapping using 785-nm laser. **a4**, **a5** Conventional LSCI (**a4**) and TR-LSCI (**a5**) for blood flow mapping using 850-nm laser. **a6** Line plots of dashed lines in (**a3**) and (**a5**). **b** Images of subcutaneous vessels in the opisthenar. **b1** photograph of the ROI indicated by the red rectangular box. **b2**, **b3** Conventional LSCI (**b2**) and TR-LSCI (**b3**) for blood flow mapping using 785-nm laser. **b4**, **b5** Conventional LSCI (**b4**) and TR-LSCI (**b5**) for blood flow mapping using 850-nm laser. **b6** Line plots of dashed lines in (**b3**) and (**b5**)

We further compared the imaging capability of TR-LSCI with conventional LSCI in human tissue, whose subcutaneous blood vessels were much deeper than that of mice. Furthermore, we demonstrated the effect of wavelength on imaging quality of TR-LSCI. As shown in Fig. 6a, when the volunteer placed the palm down over the light source and the detector was placed above the back of the hand, TR-LSCI allowed us to clearly observe blood flow in the blood vessels on the dorsal side of the finger. On the contrary, when the light source position was changed so the light diagonally illuminated the back of the palm, conventional LSCI could not provide blood flow information in the vessels. Compared with 785-nm light, the SBR of TR-LSCI image using 850 nm was better. In addition, when the volunteer rotated the hand 180°, TR-LSCI could monitor blood flow in the vessels on the ventral side of the finger, while conventional LSCI could not (Fig. S4). Figure 6b shows results of conventional LSCI and TR-LSCI for opisthenar. Similar to the finger, with conventional LSCI it was unable to obtain blood flow imaging in the vessels on the dorsal side of the palm, no matter using 785 nm or 850 nm. Furthermore, since the palm was rather thick, the TR-LSCI using 785 nm could not provide promising imaging quality, either. On the contrary, the blood flow distribution in the vessels could be monitored with 850-nm-assisted TR-LSCI. Here, the detected light intensity was kept the same for two imaging modes, thus the power intensity of TR-LSCI accessing the hand was actually higher than conventional LSCI (30 mW cm^−2^ vs. 6 mW cm^−2^). In order to exclude the influence of power density on conventional LSCI, we further improved its power density to the same as TR-LSCI, and also found it could not distinguish vessels in the human finger (Fig. S5).

**Fig. 7:**
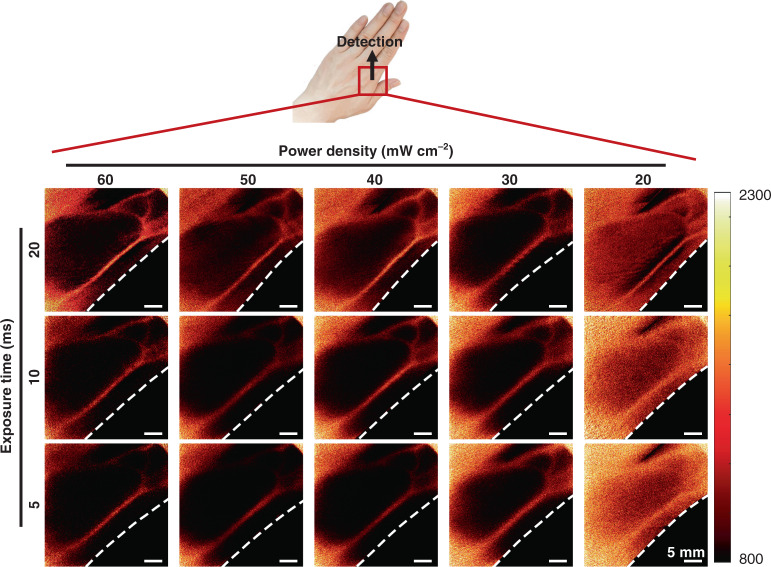
TR-LSCI for human imaging with different power densities and exposure times. Here, the light source was the 850-nm LD

Furthermore, in order to evaluate the safety of TR-LSCI on human imaging, we investigated the proper light power density as well as exposure time for TR-LSCI on human hand. As shown in Fig. 7, the results suggested the imaging quality would be acceptable when the light power density is higher than 30 mW cm^−2^. As for exposure time, the target blood vessel could be clearly distinguished even when the exposure time was 5 ms. In addition, at certain range, the imaging quality would be improved with higher power density or longer exposure time, indicating a flexible selectivity of the set parameters in the specific application, such as to decrease exposure time and increase light power density when the requirement for dynamic monitoring is relatively high. Therefore, the minimum required energy density for TR-LSCI hand imaging was only about 30 mW cm^−2^ × 5 ms/frame × 40 frame = 6 mJ cm^−2^, and even the highest energy density here (60 mW cm^−2^ × 20 ms/frame × 40 frame = 48 mJ cm^−2^) which was much lower than that used in low-level-laser therapy (usually several to dozens of J cm^−2^)^28,29^.

### Vascular functional response in human hand monitored with TR-LSCI

**Fig. 8:**
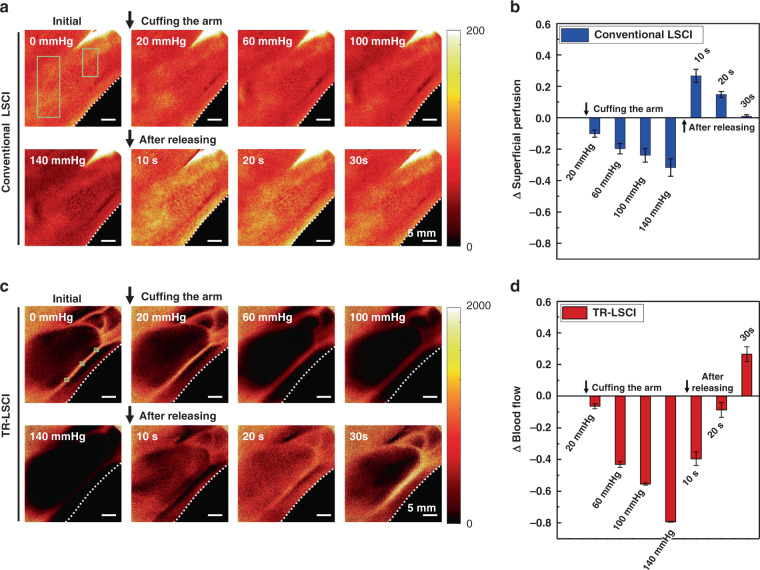
Conventional LSCI and TR-LSCI for reactive hyperemia experiments. **a** Representative conventional LSCI images of human hand at initial state, being cuffed, and after released. **b** Statistical analysis at each state based on positions indicated by the green solid line rectangular boxes in (a) was performed to show the relative changes in superficial perfusion of the hand. Data are expressed in (mean ± standard error). **c** Representative TR-LSCI images of human hand at initial state, being cuffed, and after released. **d** Statistical analysis at each state based on blood vessel positions indicated by the green solid line rectangular boxes in (c) was performed to show the relative changes in blood flow of the large vessels. Data are expressed in (mean ± standard error). Here, the light source was the 850-nm LD

Next, the ability of TR-LSCI to monitor vascular functional response in human hand was tested. Reactive hyperemia experiments were performed and the results were shown in Fig. 8. As shown in Fig. 8a, conventional LSCI showed that the perfusion in the superficial layer of the hand remarkably decreased with the increase of pressure and decreased by about 30% when the pressure increased to 140 mmHg. After release, the perfusion rapidly increased to 126% of the resting state in 10 s and eventually returned to the original state. Using TR-LSCI, the blood flow in the large blood vessels was also monitored. The result was similar to conventional LSCI, but the intensity dropped by about 80% when the pressure reached 140 mmHg, and it did not return to its resting value for 20 s after release and reached 127% of its resting value after 30 s. Such results suggested that, compared to the perfusion in the superficial layer, where there was almost microvasculature, blood flow in the deep larger vessels was more affected by pressure and recovered more slowly after pressure was released.

Other than cuffing experiments, we were also able to monitor blood flow velocity changes of human hand after exercise using TR-LSCI. As shown in Fig. S6, the blood flow response to exercise was recorded. The results indicated that the blood flow in the interested vein remarkably increased by about 0.6-fold after exercise, and began to recover after 200 s, but didn’t return to its pre-exercise level within 5 min of observation. In addition, the signal intensity in the surrounding tissue also distinguishably enhanced (Fig. 6b), which might be attributable to the acceleration of the flow velocity of capillaries and the tissue fluid.

Such results suggested that TR-LSCI provided a powerful tool for monitoring hemodynamic change in human hands.

## Discussion

We systematically evaluated the capability of TR-LSCI for thick-tissue blood flow monitoring, from Monte Carlo simulation to experimental demonstration. Dunn et al.^20^ observed that changing the position of the detector from the same side to the opposite side of the light source could improve the performance of LSCI and applied it to the acquirement of vascular functional information in human digital joints. Meisner et al.^21^ found TR-LSCI could be used to visualize blood flow in the mice hindlimb. However, neither of them has systematically explored the difference in the performance between TR-LSCI and conventional LSCI. Herein, we systematically demonstrated the potential of TR-LSCI in thick-tissue blood flow mapping. Using simulation, we clearly revealed that the ratio of the light signal from the target layer to the light signal from the whole tissue might be a significant factor that decided the imaging quality. Since the light attenuated quickly, for reflective mode, when penetrating a highly scattered tissue from the surface, the light reaching the superficial layer was much stronger and the secondary attenuation before received by the detector was much smaller, thus the imaging quality of the superficial layer could be desirable, while it would rapidly decrease when the interested layer goes deeper. On the contrary, for transmissive mode, although the secondary attenuation of the signal from the deeper layer before detected was stronger, the light irradiation was also stronger than that of the shallow layer, making the imaging quality for deep tissue also promising. Such results of simulation indicated that conventional LSCI would be superior when the ROI is in the very surface of tissue, but had shortcomings for deep signal without any further treatment to the upper layer (e.g. removing the upper tissue or make it transparent^8^), while TR-LSCI holds advantages in deep-tissue monitoring for a certain thickness of tissue.

Other than the difference between two modes on the ratio of deep signal, there might be other factors those impact the performance of conventional LSCI and TR-LSCI. For instance, LSCI is a technique that highly relies on the interference of light. However, light will become less polarized experiencing scattering events in tissue, which will be adverse to interference and so as to LSCI. It has been found through Monte Carlo simulation that compared to backward scattering, forward scattering suffers a lot much less depolarization when experiencing the same number of scattering events,^30,31^ which might suggest that TR-LSCI could hold advantage over conventional LSCI for deep detection because TR-LSCI mainly uses forward-scattered light while conventional LSCI uses backscattered light. Our study and other studies have shown that a polarizing filter would improve the performance of LSCI, which also provides support for this conjecture. In addition, further simulations and experiments should be performed to mechanically discuss the dynamic light scattering behind the TR-LSCI, like how many scattering events are expected for thick tissue, and what the correlation functions at different depths should be, similar to what was done for conventional LSCI.^24,32^ In this case, TR-LSCI can be further studied and optimized.

For the experimental study of TR-LSCI, the previous works were not systematically designed, but just chose one part of mice or human body^21^. Here, mice body parts with various thicknesses, including the ear, hindlimb, back and paw were chosen as imaging sites to evaluate the validity of our simulation, as well as the capability of TR-LSCI for monitoring the targets where structural and functional information of blood vessels is always desired by researchers in the basic research of microcirculation^7,25–27^. In addition, different positions of human hand were also investigated. Our systematical in vivo results also suggested that TR-LSCI could perform blood flow mapping with promising resolution in thick tissue without the assistance of tissue windows, those conventional LSCI needs for the research of tumor vasulature^33,34^, wound healing^35^, therapeutic effect of thrombolytic drugs^36,37^, photodynamic therapy of malformed blood vessels^38–40^ and so on.

Furthermore, the successful acquirement of blood flow signal in human hand implied that TR-LSCI might be further applied in other human body parts whose thickness is feasible for light to penetrate, such as ear, lip, toe, and instep etc. Thus, the development of TR-LSCI might accelerate the clinical research of microcirculation and related diseases, such as diabetic foot ulcer^41^, rheumatoid arthritis^42^, and dermatitis^43^. Moreover, our results showed that, 850-nm wavelength LD as imaging light provided better imaging quality than that of 785 nm, which we attributed to the greater penetration depth at 850 nm. In fact, NIR-II (900–1700 nm) light has even more promising penetration and has been widely used in deep-tissue imaging in recent years^44^. Compared to NIR-IIb (1560–1700 nm), where the high absorption of tissue might limit the application of TR-LSCI, NIR-IIa (900–1300), especially 1300 nm, has relatively low absorption and acceptable scattering in tissue. Consequently, TR-LSCI using such wavelength range might hold great potential for high-resolution blood flow mapping in further clinical use. Nevertheless, it should be noticed that a promising detector is important for human TR-LSCI imaging. Herein, using a high-end CCD that responded more than 90% in 400–900 nm wavelength range, the imaging laser power density could be set as low as 30–60 mW cm^−2^. However, it might not directly work for cameras typically used in conventional LSCI, especially in the NIR region, where it would require higher light power density for human applications.

Admittedly, TR-LSCI has its own limitations. Like conventional LSCI, TR-LSCI cannot distinguish the depth of vessels, nor allow to measure absolute blood velocity, but it is only capable of qualitative analysis of blood flow dynamics. Furthermore, although our study clearly demonstrated that, TR-LSCI could obtain high-resolution blood flow information in tissue with a certain thickness and optical properties, such as the ear, hindlimb, paw, back of mice, and human hand or perhaps other small human body parts in future, when the tissue thickness or the scattering is overlarge, it might be better to use conventional LSCI with a tissue window. For instance, for mice brain monitoring, as shown in Fig. S7, it was impossible to obtain cortical information using either LSCI, because the bones and skin above and below the brain tremendously scattered and attenuated the light. After removing the scalp and skull, since the light could directly reach to the brain, the superficial blood vessel could be clearly observed using conventional LSCI. However, for TR-LSCI, the light still needed to penetrate the skin and bones below the brain those could not be removed. Consequently, even with an open-skull window, TR-LSCI could not perform high-resolution cortical blood flow mapping.

In summary, our study revealed an alternative of LSCI for noninvasive thick-tissue blood flow imaging and discussed its proper applications with simulations and experiments. As thus, according to the specific application, a more suitable imaging scheme can be selected between conventional LSCI and TR-LSCI.

## Materials and methods

### Monte Carlo simulation

Here, Monte Carlo simulation was used to theoretically compare reflective and transmissive imaging modes in thick tissue. According to the optical characters of skin to 633-nm light^45^, the refractive index, absorption coefficient, scattering coefficient, and anisotropy factors were set as Table 2.

**Table 2:** Parameters of different skin layers used in Monte Carlo simulation

Skin layer	Refractive index (*n*)	absorption coefficient (μ_a_)/cm^−1^	scattering coefficient (μ_s_)/cm^−1^	Anisotropy factor (g)
Stratum corneum	1.50	0.15	175.0	0.90
Living epidermis	1.34	2.47	87.5	0.80
Dermis	1.40	0.28	80.6	0.82

The light source was set as an infinitely narrow photon beam, which was incident perpendicular to the tissue. Two series of simulations were performed. The first one was based on a single-component tissue (dermis, 520 μm thickness), and the second one was based on a multi-component tissue: stratum corneum (20 μm)-epidermis (80 μm)-dermis (520 μm). To simplify the model, we discussed only seven layers in the stimulated single-component tissue, those were located at 0, 100, 200, 300, 400, 500, and 520 μm, respectively, and discussed nine layers in the stimulated multiple-component tissue, those were located at 0 (the surface of stratum corneum), 20 (the surface of epidermis), 100 (the surface of dermis), 200, 300, 400, 500, 600, 620 μm, respectively.

For reflective imaging, we assumed that the light was irradiated from the top of the tissue, and the desired signal from the target layer, as well as the background signal from other layers, was detected at the top of the tissue. To calculate the ratio of the signal from the target layer to the signals from all the seven layers (here we defined it as “signal-to-background ratio, SBR”), a two-step Monte Carlo simulation was performed. Firstly, we simulated the luminous flux distribution of light as it penetrated the entire tissue, thereby obtaining the luminous flux ratio of the seven layers described above. Secondly, we set the initial light flux of each layer according to this ratio to simulate the remaining light flux when the light of each layer returning to the surface. In this case, we could obtain the ratio of the luminous flux returned from every layer, and the SBR could be calculated when any layer was set as the target layer.

The simulation and calculation of the SBR of transmissive imaging were similar, where the differences were that the light was irradiated from the bottom of the tissue, and the desired signal from the target layer as well as the background signal from other layers was detected at the top of the tissue.

To investigate the effect of the thickness above or below the target layer on the SBR of reflective- and transmissive-detected modes, we chose the target layer at 200 μm in the 520-μm-tissue as the initial situation where their SBR was approximate. Then the position of the signal layer kept constant and the thickness above or below the signal layer increased to 400 μm taking 100 μm as the step size. The simulated tissue parameters remained the same as previously mentioned and the SBR of two imaging modes when the tissue had different thicknesses was calculated, respectively.

To perform the Mento Carlo simulation corresponding to the tissue phantom experiment, we first constructed the simulated tissue of 5 mm and divided it into 11 layers those were located in 0, 0.5, 1, 1.5, 2, 2.5, 3, 3.5, 4, 4.5, 5 mm, respectively, to simulate the situation where the total thickness was constant, but the signals depth varied from 0 mm to 2.5 mm. Then we constructed another simulated tissue to simulate the situation where the position of the signal was constant and the thickness of the upper layer of the signal continuously increased. The tissue was first set as 3.5-mm thick and simplified to 8 layers located in 0, 0.5, 1, 1.5, 2, 2.5, 3, 3.5 mm. The target layer was fixed at 0 mm of such tissue, and then the upper thickness of the tissue increased by 0.5 mm every time until the whole tissue thickness was 5.5 mm.

### TR-LSCI and conventional LSCI

**Fig. 9:**
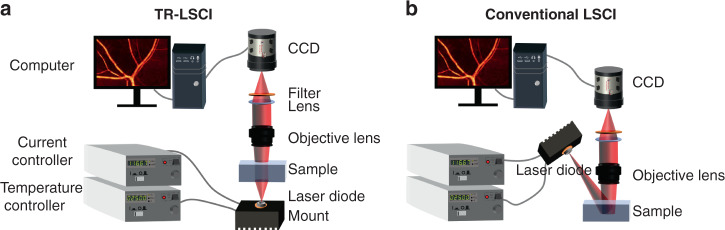
Schematic of LSCI systems. **a** Transmissive-detected LSCI system. **b** Conventional reflective-detected LSCI system.

Figure 9a, b shows the schematic of TR-LSCI and conventional LSCI systems, respectively. For TR-LSCI, a laser diode (L785P090 or L850P200, Thorlabs, USA) was installed on the mount (LDM56/M, Thorlabs, USA), and controlled by a current controller (LDC205C, Thorlabs, USA) and a temperature controller (TED220C, Thorlabs, USA). The light emitted by the laser diode illuminated beneath the sample. The beam passed through the entire sample and was collected by a stereomicroscope (SZX12, Sunny, China). On the contrary, for conventional LSCI, the LD illuminated obliquely above the sample. The laser beam was scattered by the sample surface and entered the stereomicroscope. The stereomicroscope was equipped with a filter (FL780-10: 780 ± 10 nm or FL850-10: 850 ± 10 nm, Thorlabs, USA) and a CCD (Pixis, Princeton Instrument, USA). Unless otherwise specified, the exposure time of the CCD was set as 20 ms and 40 continuous frames were captured to acquire the blood flow distribution with laser speckle temporal contrast analysis through the following equation^13^:

$${{{{K}}}}_{{{{{t}}}}_{({{{{x}}}},{{{{y}}}})}} = \frac{{{\upsigma }}_{({{{{x}}}},{{{{y}}}})}}{{ < {{{{I}}}}_{({{{{x}}}},{{{{y}}}})} > }}$$

where *K*_*t*(*x*,*y*)_ was the temporal contrast at pixel (*x*,*y*), σ_(*x,y*)_ was the standard deviation of pixel intensity corresponding to this coordinate in 40 images, and <*I*_(*x,y*)_> was the average of pixel intensity corresponding to this coordinate in 40 images. 1/*K*_*t*_^2^ could reflect the speed of blood flow. The actual imaging speckle size was determined using autocovariance function, and was adjusted by changing the aperture of the system^23^.

In order to maintain the consistency of experimental conditions in the comparison, we set the laser power according to two principles. In most of our experiments, we ensured that the detector received the same intensity of light for both transmissive and reflective imaging, which could be done through the detector readings. In this case, the laser power density irradiated on the sample of TR-LSCI was much larger than that of conventional LSCI. Consequently, we performed another additional human hand imaging experiment, in which we turned up the power density of the light on the hand for the conventional LSCI to match that for the TR-LSCI to study if it would improve the imaging quality of conventional LSCI.

### Construction and imaging of tissue phantom

As shown in Fig. 2a, in the tissue phantom, 1% intralipid (Fresenius Kabi, China) mixed with 0.5% agarose (Sigma-Aldrich, USA) gel at a volume ratio of 1:1 was chosen to simulate the turbid tissue because its reduced scattering coefficient was similar to that of in vivo skin^45^. Capillary glass tube (inner diameter: 0.5 mm, outer diameter: 1.0 mm) was used to simulate the blood vessel embedded in the turbid tissue. The capillary glass tube was insert diagonally into a culture dish (diameter: 32.1 mm, height: 10 mm). Blood was taken from the caudal venous plexus of mice and mixed with heparin sodium (Sinopharm Chemical Reagent, China) to prevent clotting. After drawing mice blood, a micro syringe was clamped on the micro syringe pump, and its needle tip was inserted into the capillary glass tube. Then, the blood was pumped into the capillary with certain velocities.

Another kind of tissue phantom was constructed by inserting the capillary glass tube into the culture dishes horizontally. The bottom of the capillary was 2.5 mm from the bottom of the dish and the thickness (3.5 mm, 4 mm, 4.5 mm, 5 mm, 5.5 mm) of the mixture of intralipid and agarose was changed by adding a mixture of different volumes to culture dishes. The capillary glass tubes in these two kinds of tissue phantom were imaged with TR-LSCI and conventional LSCI equipped with the 785-nm LD and blood flow maps were acquired. Then, we evaluated the imaging quality by subtracting background from the signal.

### Animal treatment

All animal procedures were approved by the Experimental Animal Management Ordinance of Hubei Province, China, and carried out in accordance with the guidelines for humane care of animals. Mice (Balb/c, male, 8 weeks old) were anesthetized by gas anesthesia machine (1.5% isoflurane) during experiments. The hair on ear, hindlimb, back and paw was shaved and the remaining hair was cleaned by treating with depilating cream. Mice ear, hindlimb and paw were placed directly on the experiment platform, respectively. In specific, to perform blood flow imaging in dorsal skin, two-fold skin at the shaved spot was lifted and fixed with an aluminum bracket. The raw speckle images were acquired with TR-LSCI and conventional LSCI under the irradiation of 785-nm laser.

To test the capability of LSCI to dynamically monitor the response of blood flow to drugs in mice, the blood flow of the subcutaneous blood vessels in mice hindlimb was recorded in its resting state for 160 s, followed by a vein injection of 125 μL of Ach (Sigma-Aldrich, USA, 0.2 mg mL^−1^) and a continuous recording for 11 min. We acquired the blood flow intensity in the blood flow maps at every time point with MATLAB and calculated the relative changes of blood flow (Δ Flow), which was defined as:

$${\Delta}\;{{{{Flow}}}} = \left( {{{{{I}}}}_{{{{i}}}} - {{{\mathrm{I}}}}_{{{{\mathrm{BL}}}}}} \right)/{{{{I}}}}_{{{{\mathrm{BL}}}}}$$

where *I*_*i*_ was the blood flow intensity at a certain time point and *I*_BL_ was the baseline value, that is, the average value of blood flow intensity before stimulation.

### H&E staining

To measure the thickness of tissue above vessels in each part of mice imaged by LSCI, H&E staining was performed. The entire ear as well as the skin on hindlimb, back and paw were removed from the mice. After cleaning with phosphate buffer saline, these samples were fixed in 4% neutral paraformaldehyde for 24 h and embedded in paraffin. The samples were cut transversely into sections and stained with hematoxylin & eosin. Finally, the sections were magnified 400 times and scanned with white light.

### Human data acquirement

In order to further verify the improvement of the imaging quality of thick tissue by TR-LSCI, we imaged subcutaneous vessels in human hand, which are much deeper than aforementioned body parts of mice.

Volunteers placed their fingers or palms on the experiment platform, and the raw speckle images were acquired with TR-LSCI and conventional LSCI equipped with 785-nm or 850-nm LD, respectively. In addition, we used TR-LSCI to monitor the dynamic change of blood flow in the individual blood vessel. For reactive hyperemia experiment, after acquiring blood flow imaging in the resting state, the arm of the volunteer was cuffed by a sphygmomanometer (Yuwell-Jiangsu Yuyue medical equipment & supply Co., Ltd, China) and then released, during which period the blood flow was monitored while the pressure was recorded. For squat experiment, after acquiring blood flow imaging in the resting state, the volunteers did squats for 2 min, and then we monitored the change of blood flow in the same place during the next 5 min. The calculation of relative changes of superficial perfusion and blood flow was similar to that of animal experiments.

## Supplementary information

Supplementary information
