# Supplementary material for: Transmissive-detected laser speckle contrast imaging for blood flow monitoring in thick tissue: from Monte Carlo simulation to experimental demonstration
Source: Light Sci Appl. 2021 Dec 3;10:241. doi: 10.1038/s41377-021-00682-8 (PMC8642418; doi:10.1038/s41377-021-00682-8)
Supplement: Supplementary file 1 — Supplementary information [file 41377_2021_682_MOESM1_ESM.docx]

Supplementary Information for Transmissive-detected laser speckle contrast imaging for blood flow monitoring in thick tissue: from Monte Carlo simulation to experimental demonstration

Dong-Yu Li, Qing Xia, Ting-Ting Yu, Jing-Tan Zhu, Dan Zhu


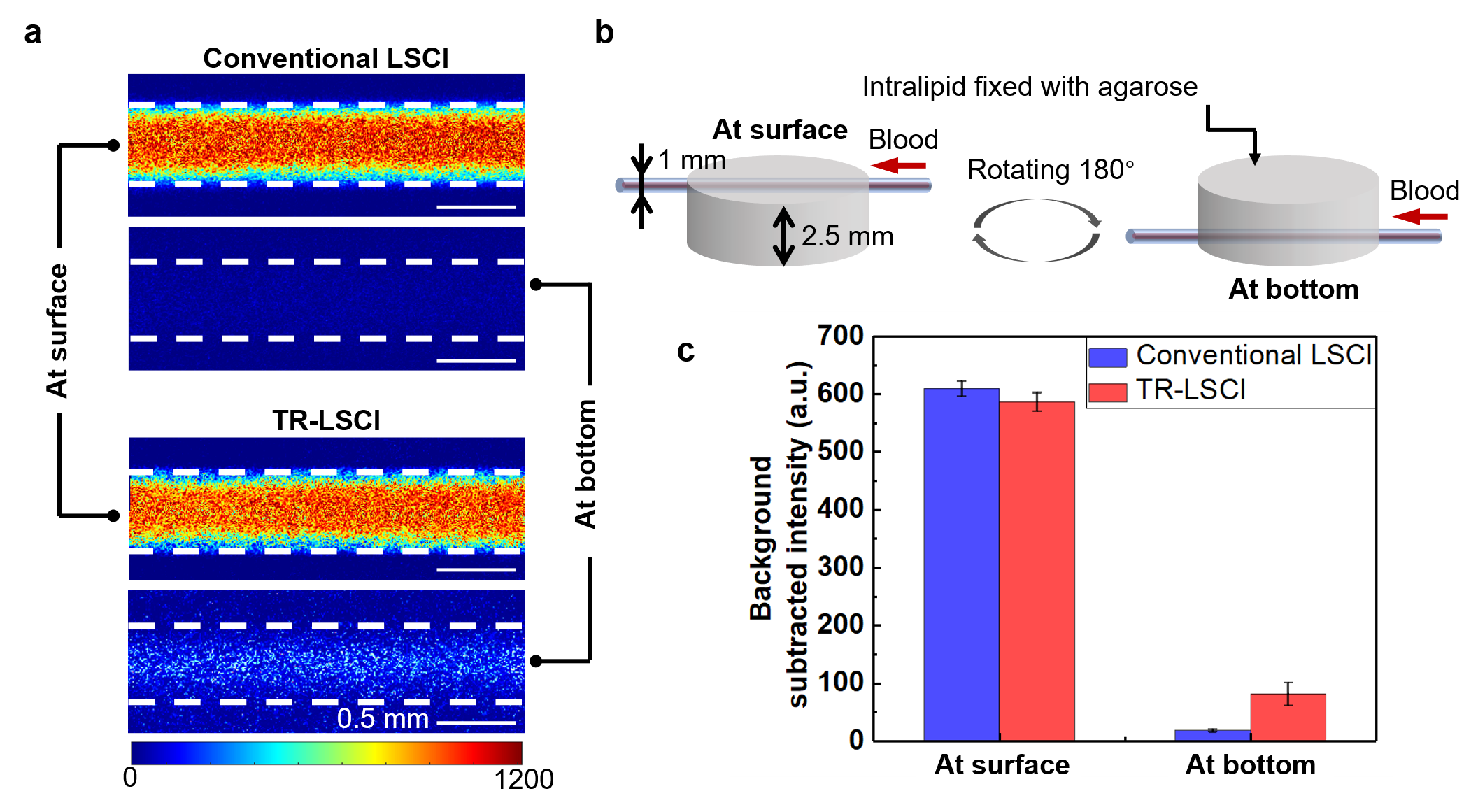


**Fig. S1 Conventional LSCI and TR-LSCI for sample rotation. a** LSCI images when the capillary was at superficial layer of the 2.5-mm intralipid or when the sample was rotated 180° as shown in (b). **c** Background subtracted signal intensity of conventional LSCI and TR-LSCI under two conditions, respectively. The blood flow velocity was set as 4 mm s^-1^. Data are expressed in (mean ± standard error).


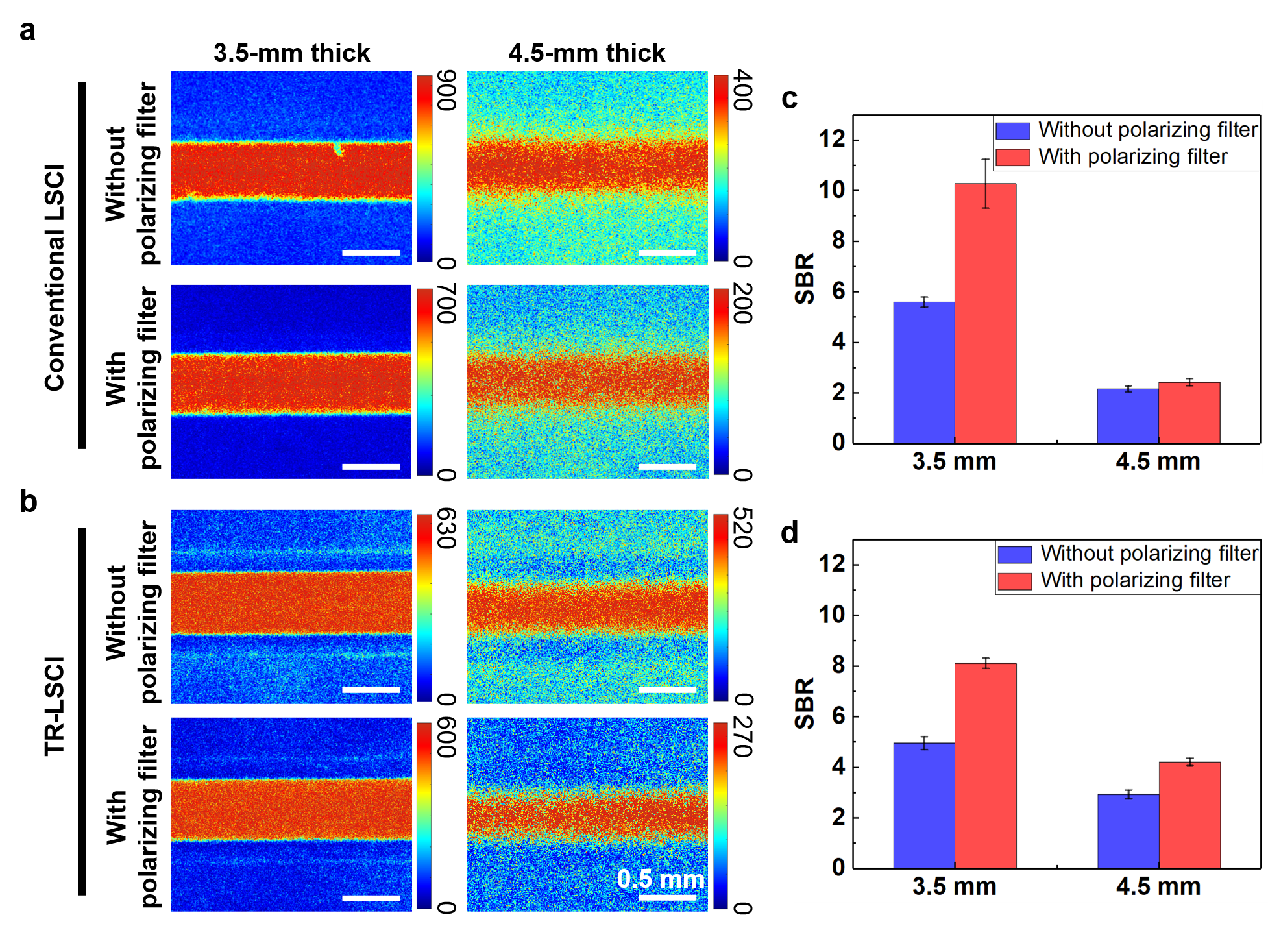


**Fig. S2 Comparison with a polarizing filter. a** Conventional LSCI images when the thickness of the intralipid was 3.5 mm and 4.5 mm, respectively. **b** TR-LSCI images when the thickness of the intralipid was 3.5 mm and 4.5 mm. **c** SBR of conventional LSCI images under each condition in (a). Data are expressed in (mean ± standard error). **d** SBR of TR-LSCI images under each condition in (b). Data are expressed in (mean ± standard error).


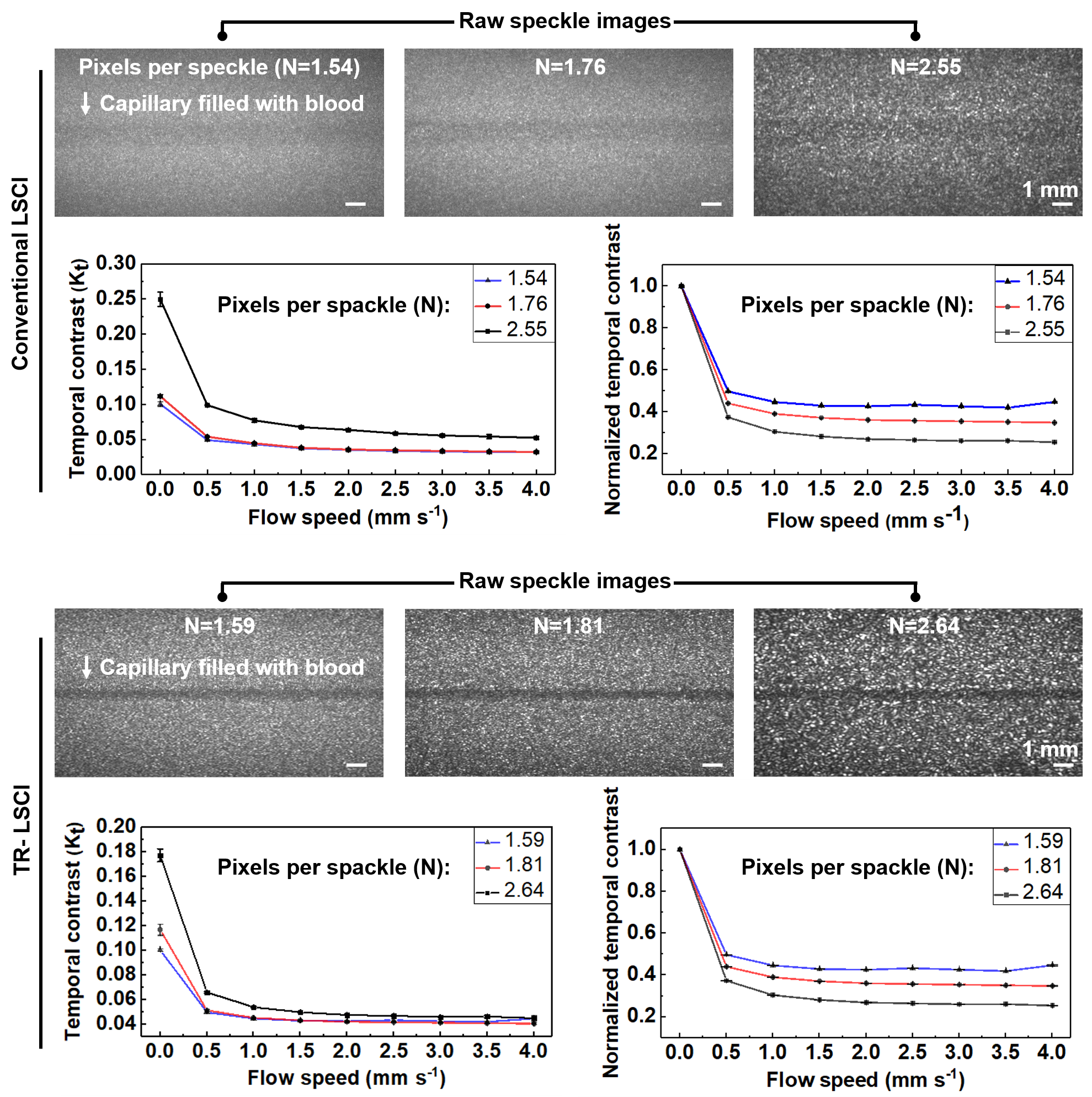


**Fig. S3 Flow change sensitivity of conventional LSCI and TR-LSCI with various speckle sizes.** The 785-nm LD was used in the experiment with 0.4× magnification. Data are expressed in (mean ± standard error).


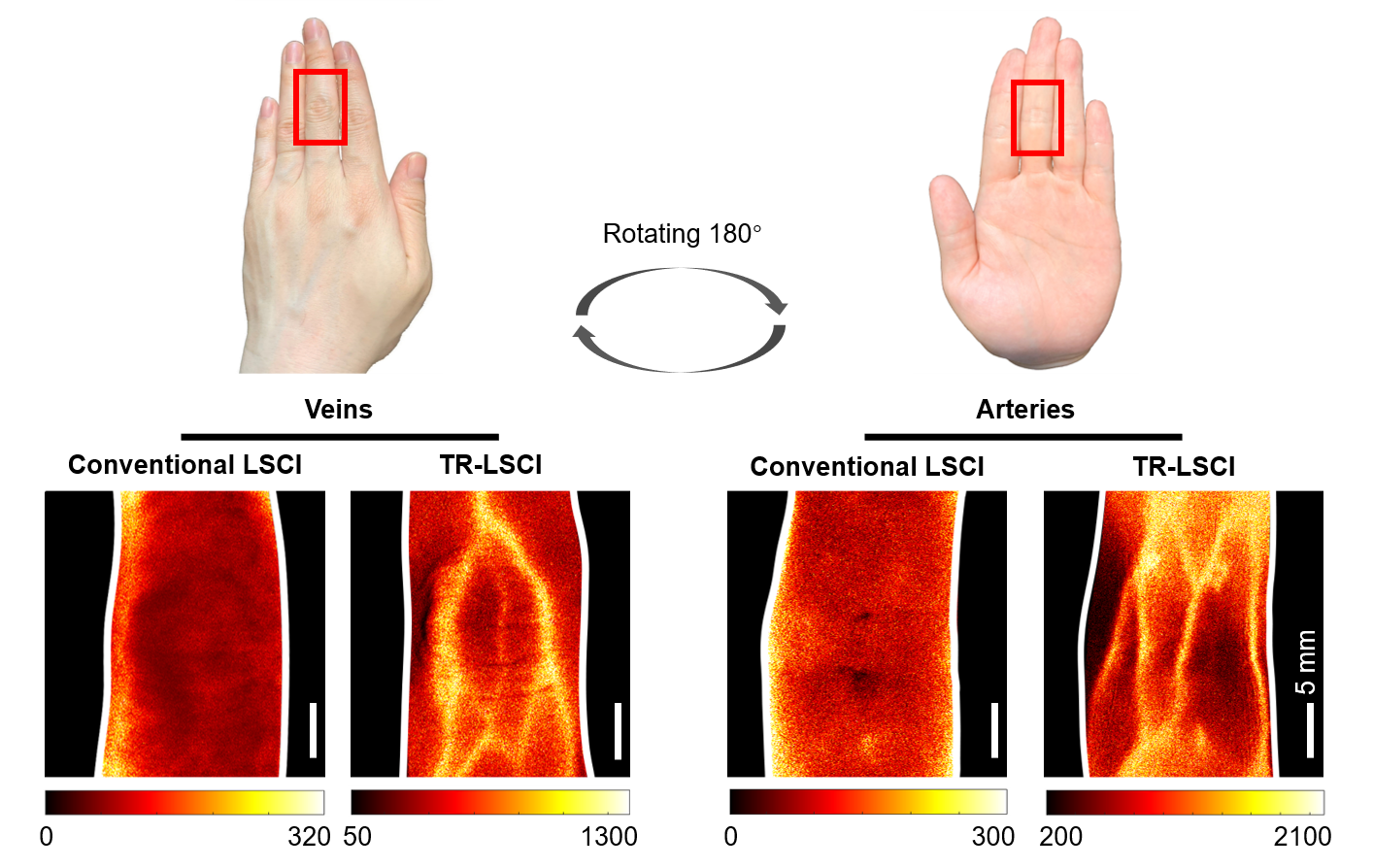


**Fig. S4 LSCI for blood mapping for dorsal and ventral blood vessels of the finger.** Here, the light source was the 850-mm LD.


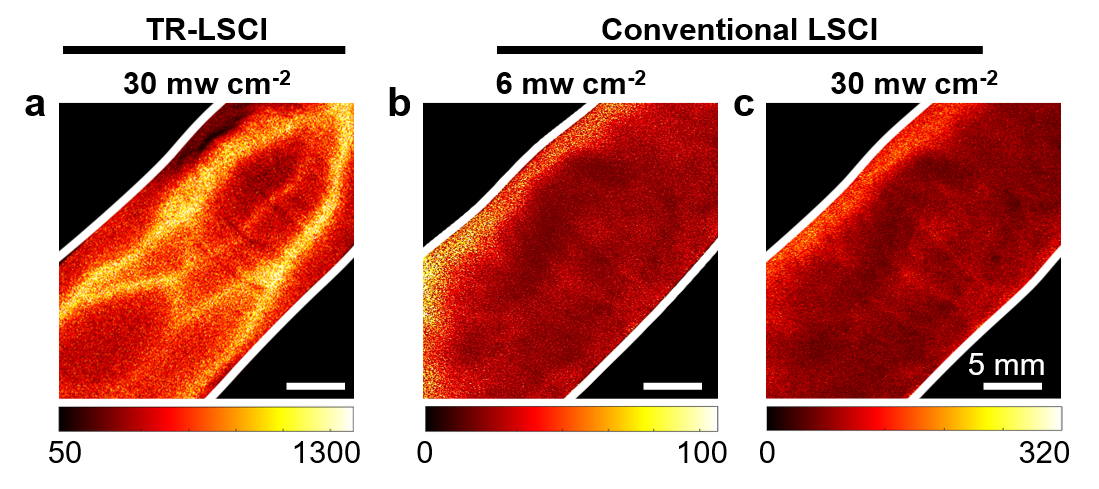


**Fig. S5 Improving light power density of conventional LSCI would not improve its imaging performance. a** The TR-LSCI image. **b** The conventional LSCI image with lower irradiated power density (to maintain that the detected light intensity was the same as TR-LSCI). **c** The conventional LSCI image with higher irradiated power density (the same as TR-LSCI). Here, the light source was the 850-mm LD.


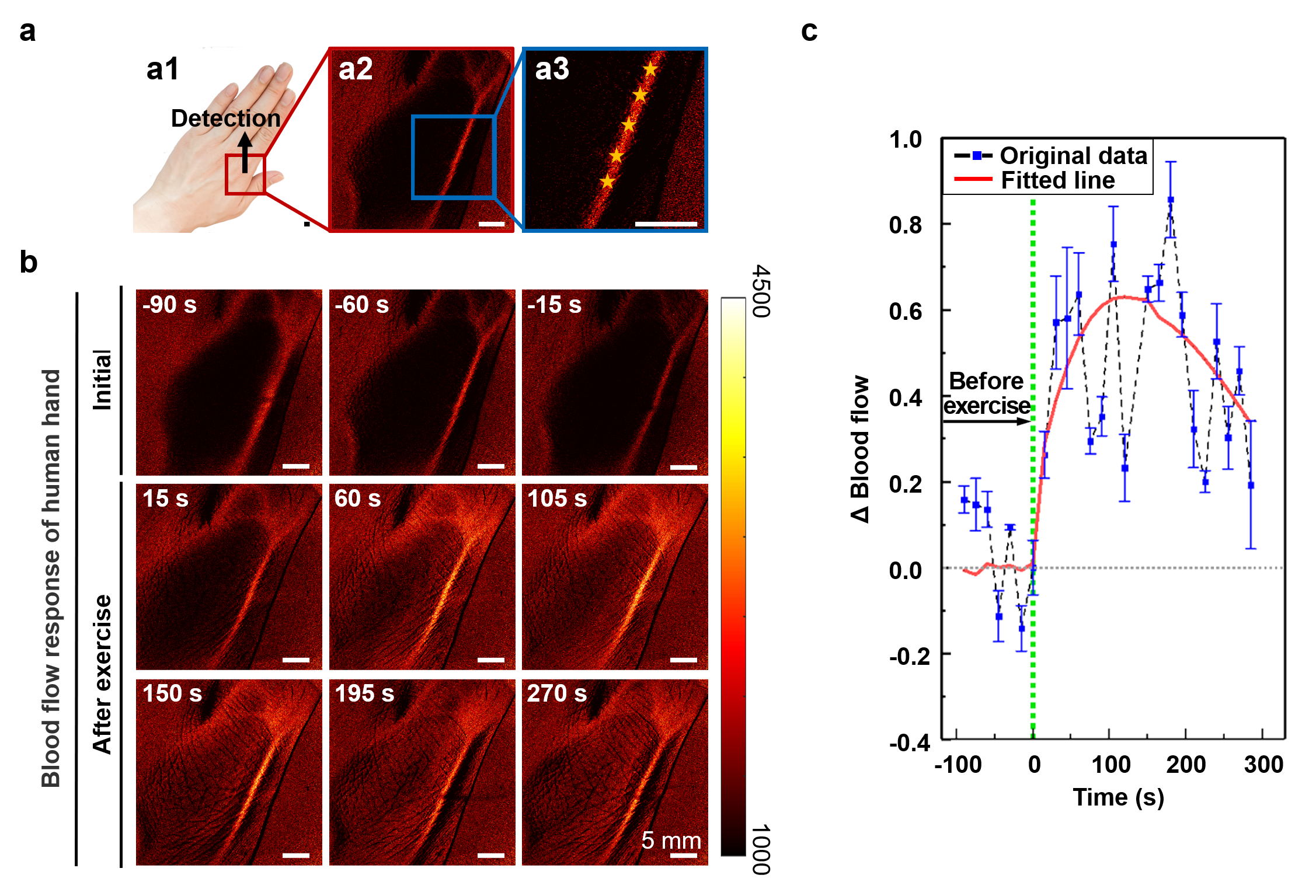


**Fig. S6** **Maps of dynamic response of blood flow in the hand of human.** **a** Images of the ROI in the hand. **a1** photograph of the hand. ROI is in the red rectangular box. **a2** Blood flow map of ROI. **a3** Amplified blood flow map in the blue rectangular box in (**a2**). Asterisks are used for statistical analysis. **b** Typical blood flow maps of subcutaneous vessels in the ROI before and after sports. **c** Statistical analysis based on vessel positions indicated by asterisks in (**a3**) was performed to show the time courses of relative changes in blood flow. Data are expressed in (mean ± standard error).


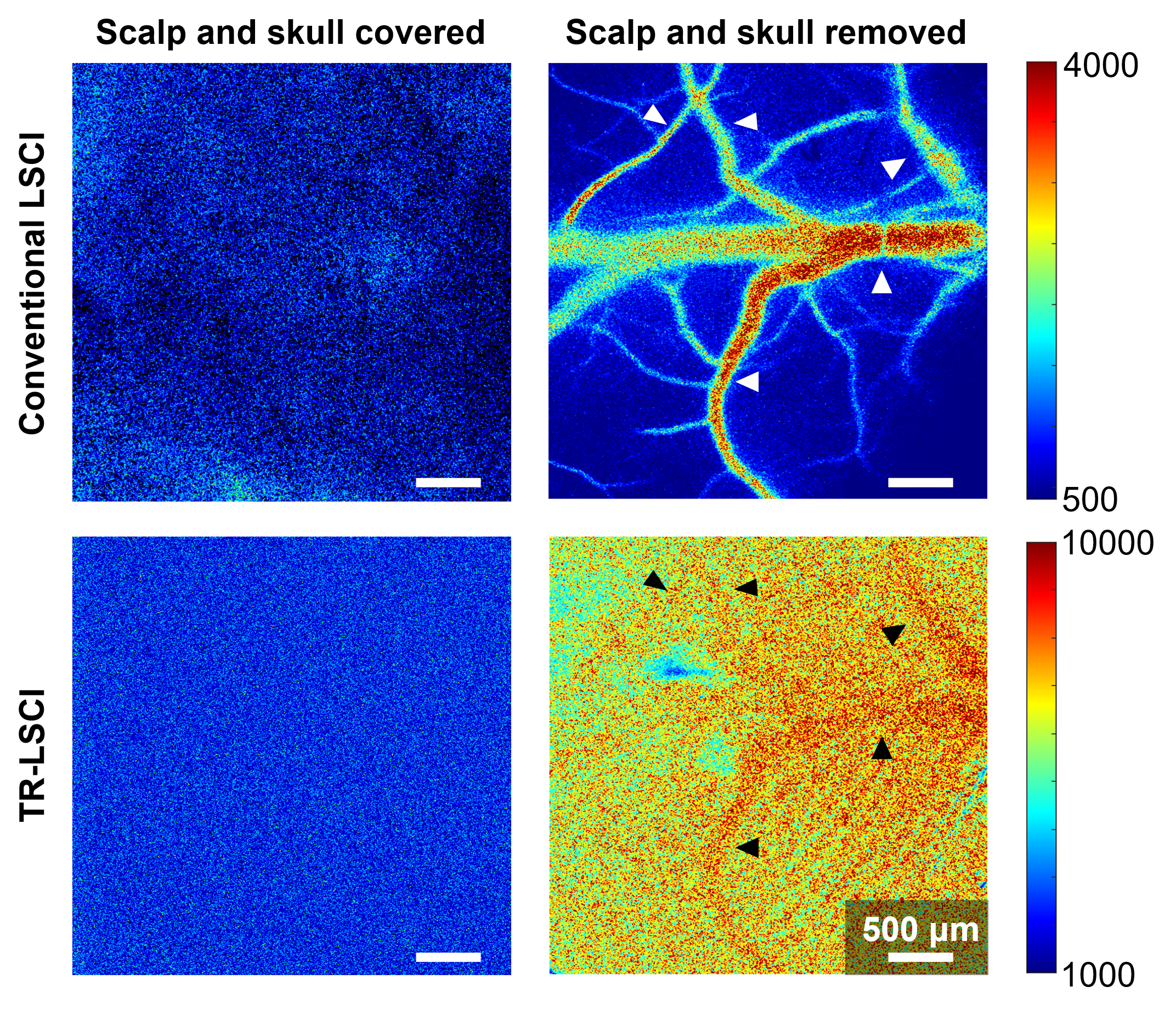


**Fig. S7 Conventional LSCI and TR-LSCI for mice cerebrovascular blood flow mapping.** The arrows indicate several blood vessels.
